# Postnatal Pancreatic Islet β Cell Function and Insulin Sensitivity at Different Stages of Lifetime in Rats Born with Intrauterine Growth Retardation

**DOI:** 10.1371/journal.pone.0025167

**Published:** 2011-10-12

**Authors:** Qingxin Yuan, Lu Chen, Cuiping Liu, Kuanfeng Xu, Xiaodong Mao, Chao Liu

**Affiliations:** Department of Endocrinology, First Affiliated Hospital of Nanjing Medical University, Nanjing, Jiangsu, China; Biomedical Research Foundation of the Academy of Athens, Greece

## Abstract

Epidemiological studies have linked intrauterine growth retardation (IUGR) to the metabolic diseases, consisting of insulin resistance, type 2 diabetes, obesity and coronary artery disease, during adult life. To determine the internal relationship between IUGR and islet β cell function and insulin sensitivity, we established the IUGR model by maternal nutrition restriction during mid- to late-gestation. Glucose tolerance test and insulin tolerance test(ITT) in vivo and glucose stimulated insulin secretion(GSIS) test in vitro were performed at different stages in IUGR and normal groups. Body weight, pancreas weight and pancreas/body weight of IUGR rats were much lower than those in normal group before 3 weeks of age. While the growth of IUGR rats accelerated after 3 weeks, pancreas weight and pancreas/body weight remained lower till 15 weeks of age. In the newborns, the fasting glucose and insulin levels of IUGR rats were both lower than those of controls, whereas glucose levels at 120 and 180 min after glucose load were significantly higher in IUGR group. Between 3 and 15 weeks of age, both the fasting glucose and insulin levels were elevated and the glucose tolerance was impaired with time in IUGR rats. At age 15 weeks, the area under curve of insulin(AUCi) after glucose load in IUGR rats elevated markedly. Meanwhile, the stimulating index of islets in IUGR group during GSIS test at age 15 weeks was significantly lower than that of controls. ITT showed no significant difference in two groups before 7 weeks of age. However, in 15-week-old IUGR rats, there was a markedly blunted glycemic response to insulin load compared with normal group. These findings demonstrate that IUGR rats had both impaired pancreatic development and deteriorated glucose tolerance and insulin sensitivity, which would be the internal causes why they were prone to develop type 2 diabetes.

## Introduction

Intrauterine growth retardation (IUGR), as the most common cause of poor fetal growth, is a common complication of pregnancy with incidence of 6.39% in China. The close relationship between poor fetal growth and many adult metabolic diseases has been confirmed by large cohorts in epidemiological studies [1]–[6]. The effects exerted by suboptimal fetal environments due to inadequate nutrition, infection, gestational diabetes, smoking or hypoxia in the mother on various systems in the offspring persist into adulthood and make the individual predisposed to metabolic diseases including insulin resistance/type 2 diabetes, hypertension, obesity, coronary artery disease, cancer and so on in the later stages of life [7]–[12].

The earliest epidemiological study linking poor fetal growth and subsequent development of type 2 diabetes was the observation by Hales et al[1], which found that among men in their 60 years, those who had lower birth weights and weights at 1 year were more likely to develop poor glucose tolerance and type 2 diabetes. Then studies from other western countries also showed us that low birth weight (less than 3 kg) had significant correlation with impaired glucose tolerance and type 2 diabetes in adulthood [2]. Recently, researches from Japan, Taiwan and India indicated that such causal relationship also existed in Asian population [4]–[6].

A theory of “Thrifty phenotype hypothesis” during critical periods early in development has been put forward to explain how early undernutrition can exert effects which persist into adulthood [13]–[15]. In human, SGA (small for gestational age) fetus was hypoinsulinemic and hypoglycemic [16], [17] and a glucose challenge in utero provoked only a small insulin secretory response [18] which persisted neonatally [19]. In the few cases which had been investigated morphologically there was evidence of a reduced pancreatic β cell mass [20]. This suggested impaired functional development of islet β cell in growth-retarded fetuses. In order to determine the internal relationship between IUGR and islet function and insulin sensitivity in detail, we established the IUGR rat model by maternal nutrition restriction during mid- to late-gestation, and selected male offsprings at different stages (newborn, 3 weeks, 7 weeks, 10 weeks, 15 weeks) in IUGR and normal groups as research objects. Intraperitoneal glucose tolerance test (IPGTT) in vivo and glucose stimulated insulin secretion (GSIS) test in vitro were performed to evaluate islet β cell function, meanwhile insulin tolerance test (ITT) were executed to learn insulin sensitivity. Therefore, this study was undertaken to investigate the internal association between IUGR and type 2 diabetes.

## Materials and Methods

### Ethics statement

All animal and tissue sample experiments were performed in accordance with the guidelines of the National Institutes of Health and Nanjing Medical University with procedures(2002-0031) approved by the Institutional Animal Care and Use Committee of the university.

### Animals

Healthy male Sprague-Dawley (SD) rats with body mass of (300±25)g and unpregnant female SD rats with body mass of (280±15)g were purchased from the Experimental Animal Center of Nanjing Medical University (Nanjing, China). All animals which were raised at Animal Lab Center Building of Nanjing Medical University were exposed to 12∶12-h light-dark cycles at 21–23°C, and had free access to water and standard rat chow.

### Maternal semistarvation model

Pregnant rats received 50% of their daily food intake beginning from day 11 through day 21 of gestation, causing caloric restriction during mid to late gestation (IUGR), compared with their control counterparts who had free access to rat chow. Both groups had ad libitum access to drinking water. The pregnant rats were allowed to deliver spontaneously, and the litter size was randomly reduced to eight at birth to assure uniformity of litter size between IUGR and control litters. The semistarvation maternal rats were provided normal diet after parturition. Two groups of pups were fostered to their mothers until they were weaned at day 21, and then all the pups were fed with standard rat chow until 15 weeks of life.

### Anthropometric measurements

Body weights were assessed longitudinally every week. In addition, at newborn, 3, 7, 10 and 15 weeks of age, the whole pancreas tissues were harvested and weighed on a scale with an accuracy of 0.001 g so as to calculate the ratio of pancreas weight to body weight (pancreas/body weight).

### Intraperitoneal glucose tolerance test (IPGTT) and insulin releasing test (IRT)

The neonatal pups from IUGR and normal group were fasted for 12 hours and then injected glucose (2 g/kg) intraperitoneally. Blood samples were collected from fossa orbitalis to determine blood glucose by an automatic glucometer (Roche, Germany) at 0, 30, 60, 120 and 180 min after the glucose load. Meanwhile, serum samples were obtained from the jugular vein for fasting insulin measurement. The samples were measured with ELISA technique (Rat Insulin ELISA kit, Linco, American).

Two groups of male rats at 3, 7, 10, 15 weeks of age were fasted overnight for 12 hours, and in the morning of the next day, blood samples were taken for determination of blood glucose and serum insulin. Glucose (2 g/kg) was injected intraperitoneally in rats. Blood samples were collected from fossa orbitalis sequentially before and 15, 30, 60, 120 min after glucose administration. Blood glucose was measured using an automatic glucometer (Roche, Germany). Serum insulin concentrations were detected via radioimmunoassay (Rat Insulin RIA kit, Linco, American). Area under the insulin releasing curve (AUCi) = 1/8×0 min insulin +1/4×15 min insulin +3/8×30 min insulin +3/4×60 min insulin +1/2×120 min insulin.

### Islet isolation and culture

Rats were anesthetized with an intraperitoneal injection of 1% pentobarbitone (50 mg/kg). After laparotomy and occlusion of the pancreatic duct near the duodenum, rat blood was drawn. The pancreas was distended by intraductal injection of 10 ml cold collagenase V (568 U/mg, Sigma, American) in D-Hanks medium. The distended pancreas was immediately excised and placed in a conical tube containing 5 ml of the cold collagenase solution. The cold ischemic time (4°C) was approximately 30 minutes. The resected pancreas was incubated in a 37–38°C water bath for 16 to 20 minutes. The pancreatic tissue was vortexed at full speed on a Vortex mixer for 3 times, 10 seconds each time. After washing 3 times with Hanks solution supplemented with 10% newborn calf serum, the undigested fragments were carefully taken out. The tissue suspension was then filtered through a 100 µm screen and washed 3 times with D-Hanks solution supplemented with 10% newborn calf serum. Islet purification was achieved by a 2-layer density gradient. The tissue pellet was resuspended in Histopaque-1077 (Pharmacia, American) and placed at the bottom. D-Hanks solution was overlaid onto the bottom layer. After centrifugation at 2200 rpm/min for 25 minutes, islets were harvested from the interface between the Histopaque-1077 and D-Hanks solution layer. After washing 2 times, the islets were handpicked and cultured at 37°C (air:CO_2_ 95∶5) in RPMI 1640 (GIBCO, American) supplemented with L-glutamine (2 mmol/l), benzylpenicillin (100 U/ml), streptomycin (0.1 mg/ml), HEPES (12.5 mmol/l) and 10% fetal bovine serum for 2 hours to recover from the isolation before starting the experimental procedures.

### Glucose stimulated insulin secretion (GSIS) test

Islets were randomly hand-picked (200∼250 um in diameter) into Milicell (Milipore, American, 20islets/well) which was placed in 24 wells plate beforehand, and preincubated in 0.5 ml basic medium, RPMI 1640 with 3.3 mmol/l glucose (Sigma, American) at 37°C (air:CO2 95∶5) for 30 minutes. After discarding the supernatants, each well was filled with 0.4 ml basic medium supplemented with 3.3 mmol/l glucose and incubated for 1 hour at 37°C (air:CO2 95∶5). The supernatants were harvested, and each well was filled with 0.4 ml stimulation medium, RPMI 1640 supplemented with 16.7 mmol/l glucose. Afterward, the 24 wells plate was incubated for another hour at 37°C (air:CO2 95∶5). The supernatants were harvested. Finally, all of the supernatant samples were stored at −20°C until insulin measurement. Insulin concentrations in the samples were assessed by Rat Insulin RIA kit (Linco, American). Stimulating index was calculated as insulin concentration in the 16.7 mmol/l glucose stimulation samples divided by insulin concentration in the 3.3 mmol/l glucose stimulation samples.

### Insulin tolerance test (ITT)

Two groups of male rats were fasted from 9AM to 3PM. For this test, 0.75unit/kg human insulin (Lilly, American) was injected intraperitoneally. Blood samples were collected via tail vein at 0, 20, 40, 60, and 90 min subsequently to measure glucose concentrations.

### Statistical analysis

Data are expressed as means ± SEM. Statistical analyses were performed using analysis of Student's unpaired *t* test (SPSS10.0). For all comparisons, the difference was considered statistically significant if *P* values<0.05.

## Results

### Growth and development status of rats at different stages of lifetime

**Figure 1:**
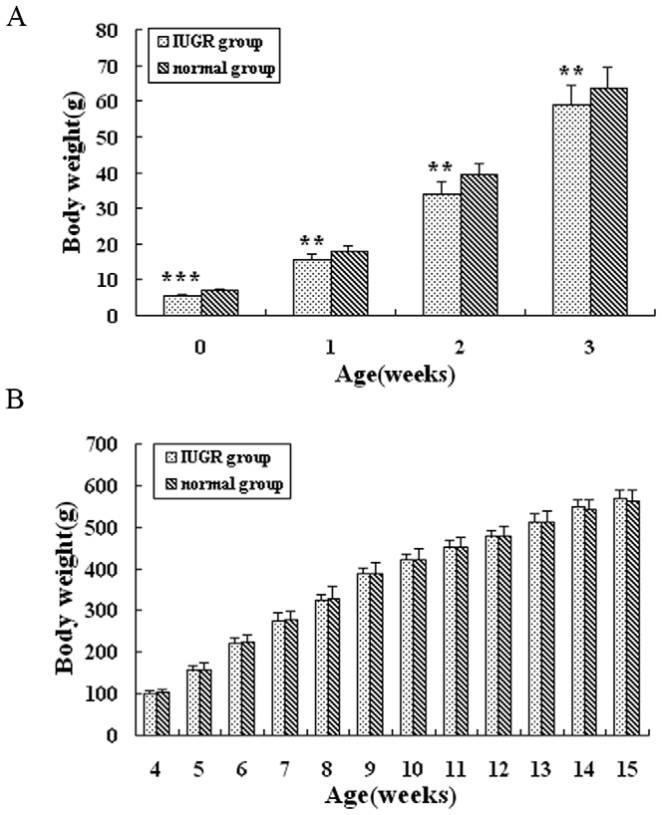
Weights of IUGR and control rats from birth until 3 weeks of age (A) and from 4 to 15 weeks of age (B). Values are the means ± SE from each group. *** *P*<0.001, ** *P*<0.01 vs. control rats.

**Figure 2:**
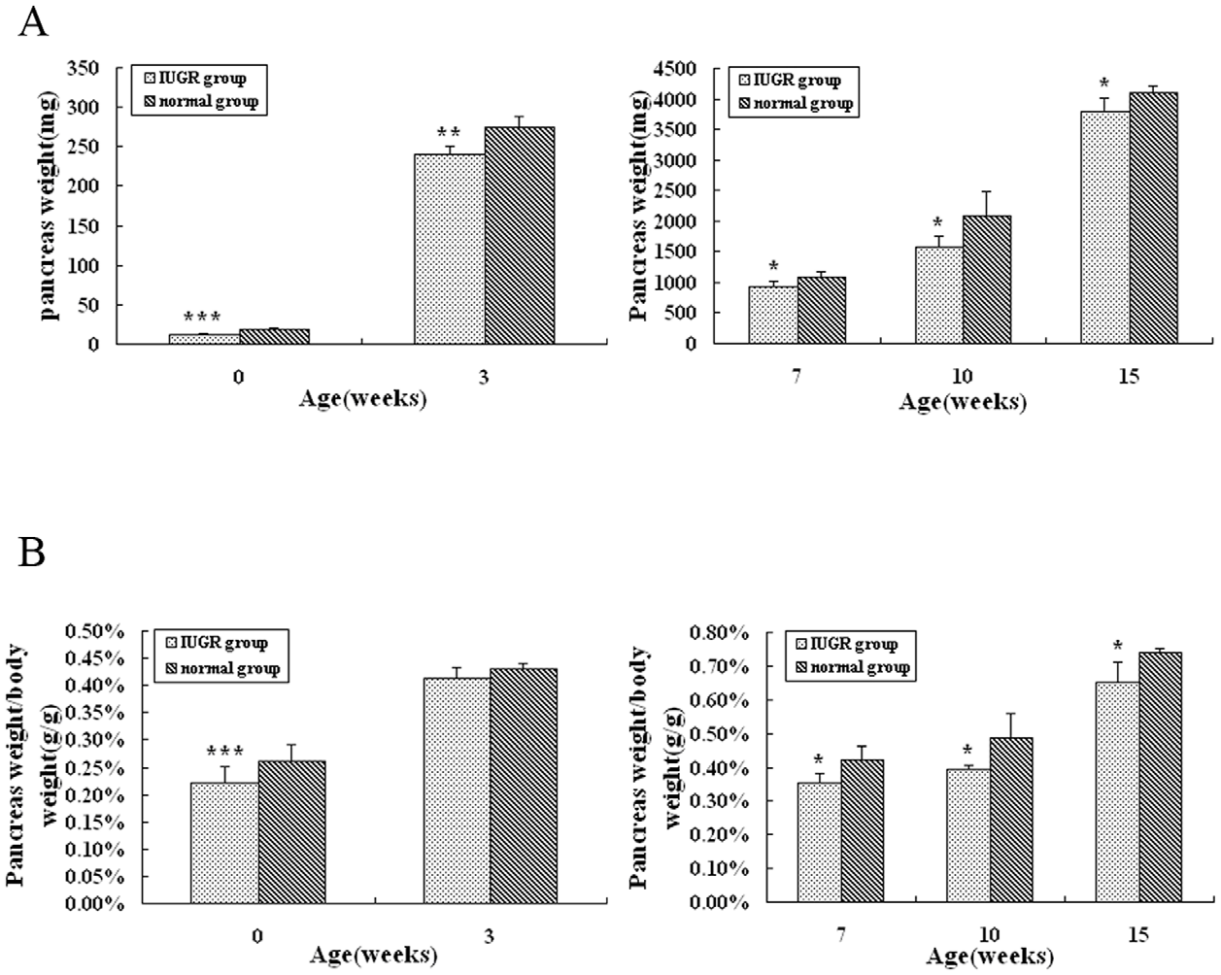
Pancreas weights (A) and pancreas weight/body weight (B) of IUGR and control rats from birth to 15 weeks of age. Values are the means ± SE from each group. *** *P*<0.001, ** *P*<0.01, * *P*<0.05 vs. control rats.

Body weights of animals from birth to 15 weeks are demonstrated (Fig. 1A and Fig. 1B). The birth weights of the offsprings born to semistarvation mothers were below the mean values of the birth weights of the control group more than 2SD, consistent with IUGR. The whole pancreas was removed and weighed at day 1, 3, 7, 10 and 15 weeks of age (Fig. 2A). Then the value of pancreas weight/body weight was calculated (Fig. 2B). Before 3 weeks of age, body weight, pancreas weight and pancreas/body weight of IUGR rats were much lower than those in normal group (*P*<0.01). While the growth of IUGR rats accelerated after 3 weeks of age and the body weight of IUGR rats even surpassed that of control at 15 weeks, pancreas weight and pancreas weight /body weight remained lower till 15 weeks of age(*P*<0.05).

### Glucose tolerance and insulin releasing

**Figure 3:**
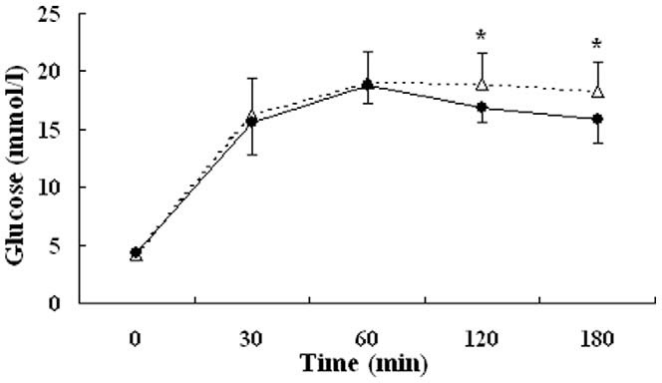
Blood glucose levels during an intraperitoneal glucose tolerance test in newborn pups in IUGR (△) and control (•) rats. Values are the means ± SE from 10 animals from each group. * *P*<0.05 vs. control rats.

To assess the function of pancreas islet β cell, glucose tolerance was performed by intraperitoneal injection of glucose. In the newborns, the fasting glucose and insulin levels of IUGR pups were both lower than those of normal ones (4.17±0.41 mmol/l vs 4.36±0.41 mmol/l; 0.20±0.02 ng/ml vs 0.25±0.07 ng/ml), whereas glucose levels at 120 and 180 min after glucose load were significantly higher in IUGR group (*P*<0.05) (Fig. 3).

**Figure 4:**
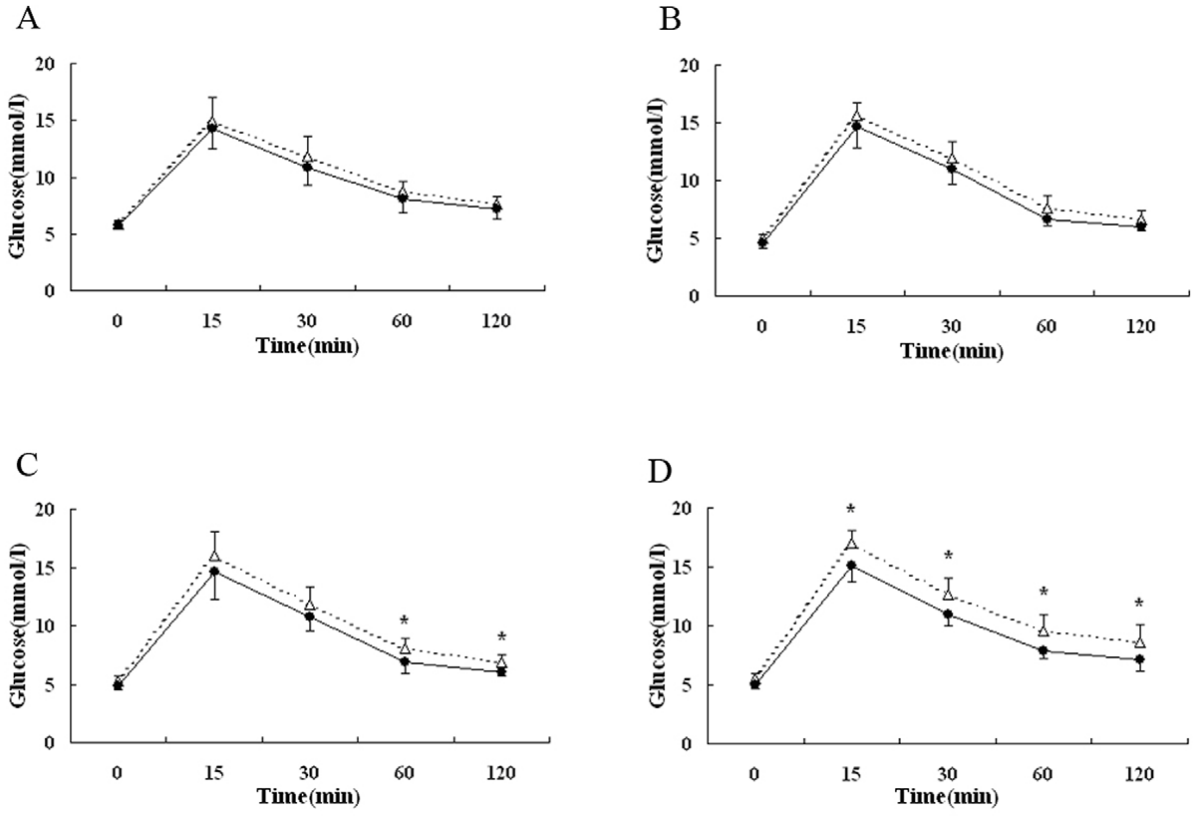
Blood glucose levels during an intraperitoneal glucose tolerance test at 3 weeks (A), 7 weeks (B), 10 weeks (C) and 15 weeks (D) of age in IUGR (△) and control (•) rats. Values are the means ± SE from 6 to 9 animals from each group at each age. * *P*<0.05 vs. control rats.

Compared with the normal control group, both the fasting glucose and insulin levels were elevated, and the glucose tolerance was impaired with time in IUGR rats from 3 to 15 weeks of age. Early in life, glucose intolerance was mild; however, as age grew, IUGR rats showed a progressive loss in the ability to handle a glucose load. At age 15 weeks, glucose levels of IUGR group at 15, 30, 60 and 120 min were significantly higher than those of control group (16.97±1.13 mmol/l vs 15.06±1.30 mmol/l; 12.61±1.38 mmol/l vs 10.96±0.92 mmol/l; 9.59±1.37 mmol/l vs 7.88±0.67 mmol/l; 8.59±1.56 mmol/l vs 7.12±0.30 mmol/l, *P*<0.05) (Fig. 4A–D).

**Figure 5:**
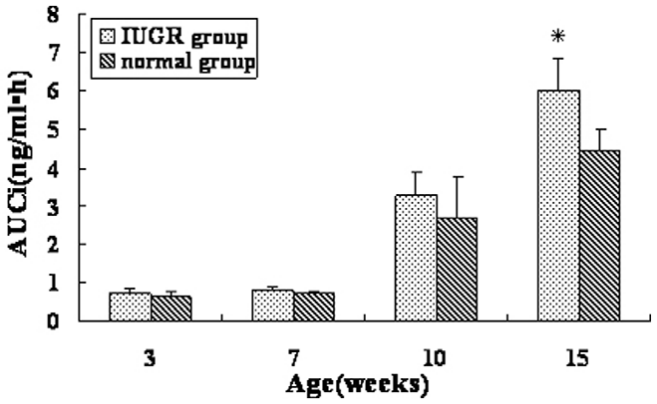
Comparison of AUCi in IUGR and control rats from 3 to 15 weeks of age. Values are the means ± SE from 4 animals from each group at each age. * *P*<0.05 vs. control rats.

In addition, insulin release was detected in response to glucose load at 3, 7, 10 and 15 weeks of age. It showed that peak insulin level was elevated and insulin concentration descended much slower in IUGR group, with higher insulin levels at 60 and 120 min after glucose administration at 15 weeks of age (3.43±0.63 ng/ml vs 2.53±0.32 ng/ml; 2.66±0.68 ng/ml vs 1.86±0.25 ng/ml, *P*<0.05) (Table 1); AUCi, considered as the area under the insulin releasing curve, was also markedly elevated (6.02±0.81 ng/ml•h vs 4.45±0.56 ng/ml•h, *P*<0.05) (Fig. 5).

**Table 1:** Serum insulin levels during IPGTT (in ng/ml).

	Time	3 weeks	7 weeks	10 weeks	15 weeks
**IUGR group**(n = 4)	**0 min**	0.17±0.06	0.24±0.04	0.51±0.17	1.10±0.66
	**15 min**	0.35±0.06	0.42±0.03	1.68±0.64	2.49±1.42
	**30 min**	0.41±0.12	0.54±0.07	2.15±0.38	3.61±0.95
	**60 min**	0.35±0.10	0.39±0.11	1.73±0.59	3.43±0.63*
	**120 min**	0.30±0.04	0.33±0.04	1.48±0.67	2.66±0.68*
**Normal group**(n = 4)	**0 min**	0.15±0.03	0.21±0.05	0.28±0.17	0.55±0.36
	**15 min**	0.33±0.07	0.38±0.09	1.46±1.52	1.74±0.71
	**30 min**	0.40±0.14	0.51±0.05	1.82±0.84	2.99±1.20
	**60 min**	0.32±0.11	0.34±0.04	1.34±0.13	2.53±0.32
	**120 min**	0.26±0.06	0.29±0.06	1.19±0.67	1.86±0.25

Comparison of insulin levels in IUGR and normal groups from 3 to 15 weeks of age. Data are means ± SE from 4 animals from each group at each age.

*P<0.05 vs. control rats.

### Insulin secretion after glucose stimulation in vitro

**Figure 6:**
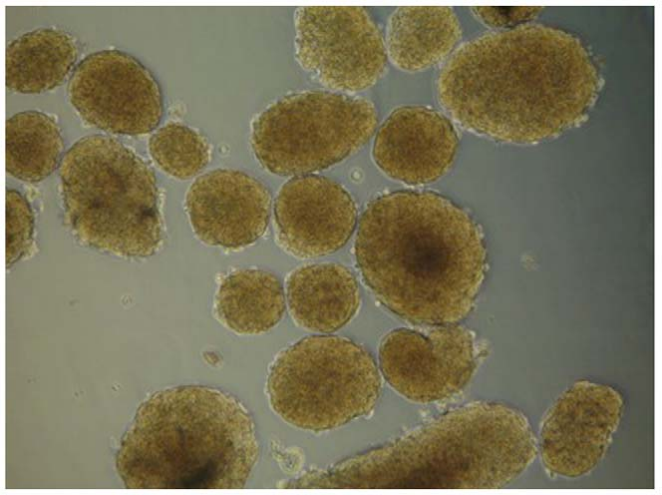
Light micrograph of rat islets. Original magnification, ×200.

**Figure 7:**
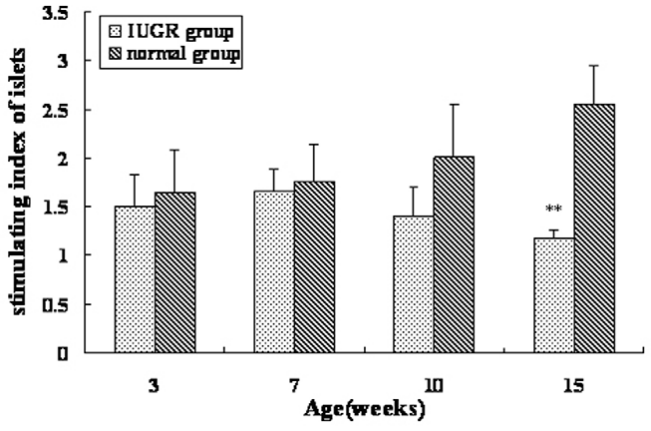
Comparison of GSIS test of islets in vitro from 3 to 15 weeks of age in IUGR and normal groups. Values are the means ± SE from 4 animals from each group. ** *P*<0.01 vs. control rats.

In order to evaluate β cell function in vitro, so as to eliminate other influencing factors, we also conducted GSIS test of islet. Under an inverted microscope, the freshly isolated islets appeared ellipse or round in shape and had a size range of 50 um to more than 500 um in diameter, with the majority measuring from 100 to 250 um (Fig. 6). The results of GSIS test of islets in vitro are shown in Fig. 7. A significant reduction of insulin release response to stimulation of 16.7 mmol/l glucose was seen in islets isolated from IUGR rats at 15 weeks of age. The stimulating index was 1.18±0.08, compared with 2.56±0.39 of control group (*P*<0.01), which indicated impaired islet secretory function in IUGR rats.

### Insulin tolerance

From 10 to 15 weeks of age, IUGR rats developed hyperinsulinemia, which suggested that insulin sensitivity of IUGR rats deteriorated with age. To determine whether this was indeed the case, we performed insulin tolerance tests. In the newborn, insulin tolerance test showed no significant difference in two groups; however, insulin sensitivity of neonatal pups was reduced compared with adult rats, no matter IUGR or normal pups. Between 3 and 7 weeks of age, there was no significant difference between two groups in insulin tolerance tests, but at age 10 weeks, IUGR rats began to show impaired insulin sensitivity. In IUGR rats, at 15 weeks, the glycemic response to exogenous insulin was a 40% drop in glucose levels after 60 min of insulin load. Although 40% drop is not a small drop in glucose levels, it has a significant difference compared to a 50% decrease in blood glucose in controls (Table 2).

**Table 2:** Serum glucose levels during ITT (in mmol/l).

	Time	Newborn (n = 7)	3 weeks (n = 5)	7 weeks (n = 6)	10 weeks (n = 5)	15 weeks (n = 5)
**IUGR group**	**0 min**	4.21±0.29	5.70±0.20	5.00±0.15	5.28±0.38	5.30±0.29
	**20 min**	5.59±0.37	4.24±0.46	4.02±0.37	4.40±0.50	4.68±0.51
	**40 min**	5.44±0.21	2.52±0.19	3.08±0.21	3.26±0.36*	3.72±0.29*
	**60 min**	5.01±0.35	1.96±0.31	2.35±0.19	2.84±0.23*	3.16±0.32*
	**90 min**	4.74±0.33	1.94±0.29	2.18±0.21	2.36±0.39	2.70±0.33*
**Normal group**	**0 min**	4.39±0.27	5.56±0.15	4.73±0.29	4.86±0.19	4.94±0.21
	**20 min**	5.67±0.42	4.06±0.47	3.72±0.28	3.84±0.28	4.12±0.27
	**40 min**	5.26±0.55	2.28±0.30	2.47±0.62	2.66±0.42	3.08±0.44
	**60 min**	4.91±0.48	1.80±0.27	2.02±0.33	2.24±0.53	2.50±0.34
	**90 min**	4.60±0.43	1.72±0.25	1.90±0.35	1.94±0.21	2.26±0.23

Comparison of glucose levels in IUGR and normal groups from newborn to 15 weeks of age. Data are means ± SE from 5 to 7 animals from each group at each age.

*P<0.05 vs. control rats.

## Discussion

In this study, we employed the mid- to late-gestation calorie-restricted IUGR rat model for this research [21], [22], which is the most critical stage for fetal growth and development [23], since nutrient restriction during the third trimester of human pregnancy is associated with the classical disproportionate IUGR. Chronic nutrient restriction may gradually alter utero-placental blood flow [21], which mimics the human condition of malnutrition existent worldwide and is also the predominant cause of IUGR in the western countries. Although IUGR may also be produced by maternal hypoxia [24] or the bilateral uterine artery ligation [25], [26], these methods will do permanent cellular damage due to compromised cellular oxidative metabolism [24] and ATP depletion [24]–[27]. Also, bilateral uterine artery ligation can result in the acute reduction of uterine blood flow so that it fails to reproduce the chronic human disease process of utero-placental insufficiency [27]. And why we chose male rats was aimed to eliminate the effect of estrogen on islet β cell function and insulin sensitivity.

In this research, the birth weights of 78.61% offsprings born to semistarvation mothers were below the mean values of the birth weights of the control group more than 2SD, which indicated IUGR rat models were made successfully. Pancreas weight and pancreas/body weight of the newborns in IUGR group were also lower than those in normal group (*P*<0.001). And as age grew, pancreas and pancreas/body weight remained lower at all stages of lifetime. It may imply that the adverse effect of a nutritionally perturbed intrauterine environment presented as early as newborn and may persist into adulthood. J Styrud et al found that in the 3-month-old rats, β cell mass was reduced by 40% in male and by 45% in female IUGR rats compared to controls via morphometric analysis of pancreas tissue [28]. All these results indicated the damage caused by utero-placental insufficiency was difficult to reverse, supposing it may has relationship with nutrient substance deficiency for islet development, intrauterine environmental failure, increased hypothalamic-pituitary-adrenal (HPA) axis activity and gene defect [29], [30].

We found that prenatal nutrient restriction and postnatal nutrient recovery would lead to a propensity toward developing obesity, that was to say, catch-up growth was present in the IUGR rats after birth in our study. Recent studies in sheep have shown that maternal nutrient restriction over the period of maximal placental growth, especially between 28 and 80 days gestation, resulted in offspring with more adipose tissue [31]. The results demonstrated that the expression of appetite regulatory neuropeptide genes in the arcuate nucleus of the hypothalamus were involved in the programming of obesity [32]. Maternal nutrient restriction would result in increased expression of orexigenic neuropeptides, while decreased expression of anorexigenic neuropeptides, thereby contributed to energy balance regulation [32], [33]. However, some researchers thought such catch-up growth was due to the increase of visceral adiposity, not proportional to the increase of skeletal muscle [34]. They supposed that offsprings restored fat as a saving approach for providing energy in a poor nutritional environment, and this adaptive mechanism would programme the development of adipose tissue, contributing to an abnormal increase of visceral adiposity [35]. Postnatal catch-up growth was also found in low birth weight children in a prospective cohort study by Ong et al [36]. A survey from Finland showed that besides low birth weight, catch-up growth between birth and 7 years of age was also an important risk factor associated with the development of insulin resistance and type 2 diabetes [37].

According to the literature, in rodents, the islets undergo substantial remodelling for about 2 weeks after birth [23]. The result of impaired glucose tolerance in normal neonatal rats in this test had a good agreement with this point of view. However, when performing glucose tolerance test on IUGR newborns, we found that though the fasting glucose and insulin levels were both lower than those of normal ones, glucose levels at 120 and 180 min after glucose load were significantly higher than normal pups, which indicated much worse glucose regulatory ability of IUGR pups. Compared with the normal group, though there was no significant difference in fasting glucose from 7 to 15 weeks of age, IUGR rats showed a progressive loss in the ability to handle a glucose load as age grew. At age 15 weeks, glucose levels of IUGR group at 15, 30, 60 and 120 min were significantly higher than those of control group, as well as abnormal releasing of insulin after glucose injection. One point we must emphasize is that we find, from this study, the abnormality in glucose metabolism of IUGR rats only presents after a glucose load, rather than fasting state.

Insulin action, as measured by insulin tolerance tests, was significantly blunted in IUGR rats at 10 and 15 weeks of age, which suggested that insulin sensitivity of IUGR rats deteriorated with age. Insulin resistance and β cell dysfunction are two characteristic features of type 2 diabetes. As showed in this experiment, islet β cell released much more insulin because of the decline of insulin sensitivity. As the insulin resistance aggravated gradually, β cell had the responsibility for the compensate of defects in insulin secretion and insulin action so as to maintain normal glucose homeostasis. However, impaired glucose tolerance progressed to overt diabetes in IUGR rat because of the ultimate inability of β cell and gradually diminished β cell mass.

In order to eliminate the interferences of insulin resistance and other insulin antagonistic hormones, we also performed GSIS test of islet in vitro to evaluate β cell function, which is known as the reliable technique for function study of primary islet accepted internationally [38], [39]. Until recently, studies of IUGR models focus on animal level in vivo, while studies in vitro has not been reported. Based on the stable technologic platform of islets isolation and purification, we successfully obtained islets of good function in IUGR rats from 3 to 15 weeks of age and detected islet secretory function in vitro. In 15-week-old IUGR rats, the relative islet stimulating index was 50% that of controls, indicated obvious deterioration of β cell function, which was also consistent with the results in vivo.

In conclusion, the data presented in our research support the “Thrifty phenotype hypothesis” that changes in the intra-uterine nutritional environment cause alterations in the islet β cell function and insulin sensitivity which have life-long effects and predispose the animal to glucose intolerance and diabetes in later life. We will have to study further and for longer time intervals in order to investigate whether the IUGR rats will develop overt diabetes later in their lifetime.

## References

[1] Hales CN, Barker DJ, Clark PM, Cox LJ, Fall C (1991). Fetal and infant growth and impaired glucose tolerance at age 64.. BMJ.

[2] Iliadou A, Cnattingius S, Lichtenstein P (2004). Low birthweight and Type 2 diabetes: a study on 11162 Swedish twins.. Int J Epidemiol.

[3] Green AS, Rozance PJ, Limesand SW (2010). Consequences of a compromised intraute- rine environment on islet function.. J Endocrinol.

[4] Anazawa S, Atsumi Y, Matsuoka K (2003). Low birth weight and development of type 2 diabetes in a Japanese population.. Diabetes Care.

[5] Simmons RA (2009). Developmental origins of adult disease.. Pediatr Clin North Am.

[6] Yajnik CS (2004). Early life origins of insulin resistance and type 2 diabetes in India and other Asian countries.. J Nutr.

[7] Fernandez-Twinn DS, Ozanne SE (2006). Mechanisms by which poor early growth programs type-2 diabetes, obesity and the metabolic syndrome.. Physiol Behav.

[8] Kanaka-Gantenbein C (2010). Fetal origins of adult diabetes.. Ann N Y Acad Sci.

[9] Shahkhalili Y, Moulin J, Zbinden I, Aprikian O, Macé K (2010). Comparison of two models of intrauterine growth restriction for early catch-up growth and later development of glucose intolerance and obesity in rats.. Am J Physiol Regul Integr Comp Physiol.

[10] Leong NM, Mignone LI, Newcomb PA, Titus-Ernstoff L, Baron JA (2003). Early life risk factors in cancer: the relation of birth weight to adult obesity.. Int J Cancer.

[11] Shen Q, Xu H, Wei LM, Chen J, Liu HM (2011). Intrauterine growth restriction and postnatal high-protein diet affect the kidneys in adult rats.. Nutrition.

[12] Pallotto EK, Kilbride HW (2006). Perinatal outcome and later implications of intrauterine growth restriction.. Clin Obstet Gynecol.

[13] Hales CN, Barker DJ (1992). Type 2(non-insulin-dependent) diabetes mellitus: the thrifty phenotype hypothesis.. Diabetologia.

[14] Hales CN, Barker DJ (2001). The thrifty phenotype hypothesis.. Br Med Bull.

[15] Ling C, Groop L (2009). Epigenetics: a molecular link between environmental factors and type 2 diabetes.. Diabetes.

[16] Setia S, Sridhar MG, Bhat V, Chaturvedula L, Vinayagamoorti R (2006). Insulin sensitivity and insulin secretion at birth in intrauterine growth retarded infants.. Pathology.

[17] Béringue F, Blondeau B, Castellotti MC, Bréant B, Czernichow P (2002). Endocrine pancreas development in growth-retarded human fetuses.. Diabetes.

[18] Nicolini U, Hubinont C, Santolaya J, Fisk NM, Rodeck CH (1990). Effects of fetal intravenous glucose challenge in normal and growth retarded fetuses.. Horm Metab Res.

[19] Yuan QX, Zhou JY, Teng LP, Xu KF, Liu C (2010). Intrauterine growth retardation leads to the functional change of insulin secretion in the newborn rats.. Horm Metab Res.

[20] Schwitzgebel VM, Somm E, Klee P (2009). Modeling intrauterine growth retardation in rodents: Impact on pancreas development and glucose homeostasis.. Mol Cell Endocrinol.

[21] Ahokas RA, Reynolds SL, Anderson GD, Lipshitz J (1986). Catecholamine- mediated reduction in uteroplacental blood flow in the diet-restricted, term-pregnant rat.. J Nutr.

[22] Thamotharan M, Shin BC, Suddirikku DT, Thamotharan S, Garg M (2005). GLUT4 expression and subcellular localization in the intrauterine growth-restricted adult rat female offspring.. Am J Physiol Endocrinol Metab.

[23] Fowden AL, Hill DJ (2001). Intra-uterine programming of the endocrine pancreas.. Br Med Bull.

[24] De Grauw TJ, Myers RE, Scott WJ (1986). Fetal growth retardation in rats from different levels of hypoxia.. Biol Neonate.

[25] Peterside IE, Selak MA, Simmons RA (2003). Impaired oxidative phosphorylation in hepatic mitochondria in growth-retarded rats.. Am J Physiol Endocrinol Metab.

[26] Sadiq HF, deMello DE, Devaskar SU (1998). The effect of intrauterine growth restriction upon fetal and postnatal hepatic glucose transporter and glucokinase proteins.. Pediatr Res.

[27] Lane RH, Ramirez RJ, Tsirka AE, Kloesz JL, McLaughlin MK (2001). Uteroplacental insufficiency lowers the threshold towards hypoxia-induced cerebral apoptosis in growth-retarded fetal rats.. Brain Res.

[28] Styrud J, Eriksson UJ, Grill V, Swenne I (2005). Experimental intrauterine growth retardation in the rat causes a reduction of pancreatic B-cell mass, which persists into adulthood.. Biol Neonate.

[29] Bréant B, Gesina E, Blondeau B (2006). Nutrition, glucocorticoids and pancreas development.. Horm Res.

[30] Langley-Evans SC (1997). Hypertension induced by foetal exposure to a maternal low-protein diet, in the rat, is prevented by pharmacological blockade of maternal glucocorticoid synthesis.. J Hypertens.

[31] Bispham J, Gardner DS, Gnanalingham MG, Stephenson T, Symonds ME (2005). Maternal nutritional programming of fetal adipose tissue development: differential effects on messenger ribonucleic acid abundance for uncoupling proteins and peroxisome proliferator-activated and prolactin receptors.. Endocrinology.

[32] Mühlhäusler BS, Adam CL, Marrocco EM, Findlay PA, Roberts CT (2005). Impact of glucose infusion on the structural and functional characteristics of adipose tissue and on hypothalamic gene expression for appetite regulatory neuropeptides in the sheep fetus during late gestation.. J Physiol.

[33] Williams G, Bing C, Cai XJ, Harrold JA, King PJ (2001). The hypothalamus and the control of energy homeostasis: different circuits, different purposes.. Physiol Behav.

[34] De Blasio MJ, Gatford KL, Robinson JS, Owens JA (2007). Placental restriction of fetal growth reduces size at birth and alters postnatal growth, feeding activity, and adiposity in the young lamb.. Am J Physiol Regul Integr Comp Physiol.

[35] Osmond C, Barker DJ (2000). Fetal, infant and childhood growth are predictors of coronary heart disease, diabetes and hypertension in adult men and women.. Environ Health Perspect.

[36] Ong KK, Ahmed ML, Emmett PM, Preece MA, Dunger DB (2000). Association between postnatal catch-up growth and obesity in childhood: prospective cohort study.. BMJ.

[37] Forsén T, Eriksson J, Tuomilehto J, Reunanen A, Osmond C (2000). The fetal and childhood growth of persons who develop type 2 diabetes.. Ann Intern Med.

[38] Liu CP, Xu KF, Liu C (2007). Isolation, purification and functional analyses of rat islets.. Acta universitatis medicinalis Nanjing (Natural Science).

[39] Liu X, Günther L, Drognitz O, Neeff H, Adam U (2006). Persistent normoglycemia in the streptozotocin-diabetic rat by syngenic transplantation of islets isolated from a single donor with Liberase.. Pancreas.

